# Challenges and Prospects in the Development of a Universal SARS-CoV-2 Vaccine

**DOI:** 10.3390/vaccines14020173

**Published:** 2026-02-13

**Authors:** Kacper Karczmarzyk, Małgorzata Kęsik-Brodacka

**Affiliations:** Department of Biomedical Research, National Medicines Institute, Chełmska 30/34, 00-725 Warsaw, Poland; m.kesik@nil.gov.pl

**Keywords:** universal vaccine, SARS-CoV-2, cross-neutralization, pan-sarbecovirus vaccines, next-generation vaccines, conserved epitopes, nanoparticles, mucosal immunity

## Abstract

The development of a universal SARS-CoV-2 vaccine holds great promise for achieving broad and durable protection against existing and future coronavirus variants. The identification, selection, and rational redesign of conserved viral epitopes constitute the direct immunological foundation of universal SARS-CoV-2 vaccine development. The breadth and durability of protection are therefore primarily determined at the level of antigen and epitope design, whereas adjuvants, delivery platforms, and routes of administration serve as enabling and amplifying components rather than primary drivers of universality. Accordingly, this review discusses key determinants of universal vaccine design, including antigen selection, adjuvant utilization, and route of administration. The spike protein, particularly its receptor-binding domain, is a major antigenic target, but its high mutation rate challenges long-term vaccine efficacy. Strategies focusing on conserved epitopes in antigen designs show potential to elicit cross-neutralizing immune responses. Nanoparticle-based vaccines capable of presenting multiple homologous or heterologous antigens have demonstrated enhanced immunogenicity, broad protection in preclinical models and safety in clinical trials. The addition of next-generation adjuvants further amplifies humoral and cellular immunity beyond the capabilities of traditional aluminum-based adjuvants. Moreover, mucosal vaccine delivery may provide superior local protection at viral entry sites and limit transmission. Importantly, integrating these technological advances with epitope-centered antigen design and immunological data from vaccinated individuals will accelerate the identification of conserved epitopes and inform future vaccine design. A multidisciplinary approach combining optimized antigen engineering, novel adjuvant systems, and innovative delivery strategies is essential for the realization of a broadly protective universal SARS-CoV-2 vaccine.

## 1. Introduction

Vaccines are among the most effective medical interventions for preventing infectious diseases, as they induce pathogen-specific immune responses that reduce morbidity and mortality at both individual and population levels [1]. The widespread implementation of vaccination programs has led to the near elimination or complete eradication of several severe infectious diseases, most notably smallpox, which caused approximately 300 million deaths in the 20th century [2]. Globally, vaccination is estimated to prevent around 2.5 million deaths annually [3]. Beyond individual protection, vaccines contribute to herd immunity, reduce the burden on healthcare systems, and play a critical role in controlling epidemics and pandemics [1]. Despite these successes, vaccine development remains challenging for rapidly evolving viral pathogens. Viruses, particularly RNA viruses, continuously accumulate mutations that can lead to the emergence of variants with increased transmissibility and reduced susceptibility to pre-existing immunity. As a result, vaccines based on earlier viral strains may show diminished effectiveness against newly circulating variants. This evolutionary pressure necessitates continuous vaccine improvement, as well as the development of safer and more adaptable vaccine platforms, such as next-generation subunit vaccines [1].

Severe acute respiratory syndrome coronavirus 2 (SARS-CoV-2) exemplifies these challenges. Although currently approved vaccines have significantly reduced COVID-19-associated transmission, hospitalizations, and mortality, their protective efficacy against symptomatic infection declines as new variants emerge [4,5,6,7]. The accumulation of mutations, particularly within the spike protein, has facilitated immune escape and increased the incidence of breakthrough infections, even in vaccinated individuals [8,9]. Consequently, the need for frequent vaccine updates highlights the limitations of strain-specific, spike-based vaccine strategies.

SARS-CoV-2 is the third highly pathogenic human coronavirus to emerge in the 21st century, following severe acute respiratory syndrome coronavirus (SARS-CoV) in 2002 and Middle East respiratory syndrome coronavirus (MERS-CoV) in 2012. This recurring pattern strongly suggests that additional zoonotic coronaviruses may emerge in the future, potentially triggering new outbreaks, epidemics, or even pandemics. While the rapid development of vaccines tailored to the currently circulating variants may appear to be a rational response, the time required for their production and global distribution often allows new variants to arise before such vaccines can be widely deployed [10,11].

These limitations underscore the urgent need for broadly protective vaccination strategies. It may be beneficial to develop and stockpile a pan-coronavirus (universal) vaccine that could provide at least partial protection against all viruses within the Betacoronavirus genus, including the subgenera Sarbecovirus (e.g., SARS-CoV and SARS-CoV-2) and Merbecovirus (e.g., MERS-CoV), rather than waiting for the next novel coronavirus to appear.

This review is based on a focused narrative analysis of the current literature addressing universal and pan-coronavirus vaccine development. Relevant publications were identified through searches of PubMed, Scopus, and selected preprint servers (including bioRxiv and medRxiv), using combinations of keywords such as “universal coronavirus vaccine,” “pan-coronavirus vaccine,” “SARS-CoV-2 variants,” “conserved epitopes,” “nanoparticle vaccines,” “mucosal vaccination,” and “T-cell-based vaccines.” The literature surveyed primarily covers the period from 2020 to early 2025 and includes both preclinical and clinical studies, with emphasis on vaccine strategies targeting SARS-CoV-2 and related sarbecoviruses. Studies were selected based on their relevance to antigen design, immune mechanisms, breadth of protection, and translational potential.

Importantly, the achievable breadth and durability of protection of any universal coronavirus vaccine are ultimately defined at the level of antigen and epitope selection, whereas vaccine platforms, adjuvants, and routes of administration function primarily as secondary modifiers of immunogenicity rather than determinants of universality.

On this basis, this review focuses on the key challenges and prospects associated with the development of a universal coronavirus vaccine.

## 2. Rationale, Design Principles and Necessity of a Universal SARS-CoV-2 Vaccine

### 2.1. Conceptual Scope and Taxonomy of Universal and Pan-Coronavirus Vaccines

A universal vaccine is conceptually defined as a vaccine designed to provide broad protection against a pathogen despite ongoing antigenic evolution, enabling rapid deployment and preparedness in the event of future outbreaks or pandemics [12]. In practice, however, the term “universal vaccine” has been used inconsistently in the literature and often refers to formulations that induce immune responses against only a limited number of viral strains rather than truly variant-independent protection. To improve conceptual clarity and facilitate meaningful comparison between vaccine strategies, it is therefore important to distinguish the aspirational goal of universality from the operational breadth of protection achieved by a given vaccine candidate.

In this review, the term universal vaccine is used to denote the overarching objective of achieving durable and broadly protective immunity, whereas specific vaccine strategies and candidates are categorized according to their pan-X spectrum, defined by the phylogenetic range of viruses they are designed to cover. Pan-variant vaccines aim to protect against all circulating and future variants of SARS-CoV-2. Pan-sarbecovirus vaccines extend protection across the *Sarbecovirus* subgenus, including SARS-CoV and SARS-CoV-2. Pan-betacoronavirus vaccines target the broader *Betacoronavirus* genus, encompassing viruses such as MERS-CoV, HCoV-OC43, and HCoV-HKU1. More ambitious strategies include pan-subfamily vaccines directed against *Orthocoronavirinae* and, ultimately, pan-coronavirus vaccines spanning the entire *Coronaviridae* family [13] (Box 1).

Box 1 **Taxonomic Framework for Universal and Pan-Coronavirus Vaccines.**

Universal vaccine

A conceptual objective referring to vaccines designed to provide durable, broadly protec-tive immunity despite ongoing viral evolution.

Pan-variant vaccine

A vaccine designed to protect against all current and future variants of SARS-CoV-2.

Pan-sarbecovirus vaccine

A vaccine providing protection across the *Sarbecovirus* subgenus, including SARS-CoV, SARS-CoV-2, and related zoonotic viruses.

Pan-betacoronavirus vaccine

A vaccine targeting multiple viruses within the *Betacoronavirus* genus, such as SARS-CoV, MERS-CoV, HCoV-OC43, and HCoV-HKU1.

Pan-subfamily vaccine

A vaccine directed against the *Orthocoronavirinae* subfamily, encompassing both alpha- and betacoronaviruses.

Pan-coronavirus vaccine

An aspirational vaccine strategy aiming to protect against all members of the *Coronaviridae* family.

This hierarchical framework provides a practical and consistent taxonomy for evaluating vaccine candidates discussed throughout this review. Importantly, it also reflects the realistic progression of vaccine development, from near-term and achievable goals, such as pan-variant and pan-sarbecovirus protection, toward longer-term, aspirational objectives. The repeated zoonotic emergence of highly pathogenic coronaviruses, including SARS-CoV in 2002, MERS-CoV in 2012, and SARS-CoV-2 in 2019, strongly suggests that future spillover events are likely. Consequently, reliance on strain-specific vaccine updates alone is unlikely to provide sustainable global protection [10,11].

Importantly, this taxonomic framework also implies a hierarchy of vaccine design priorities, whereby epitope conservation and functional constraint constitute the primary determinants of immunological breadth, while formulation and delivery strategies modulate, but cannot substitute for, rational antigen design.

### 2.2. Desired Functional and Societal Features of a Universal Coronavirus Vaccine

**Figure 1:**
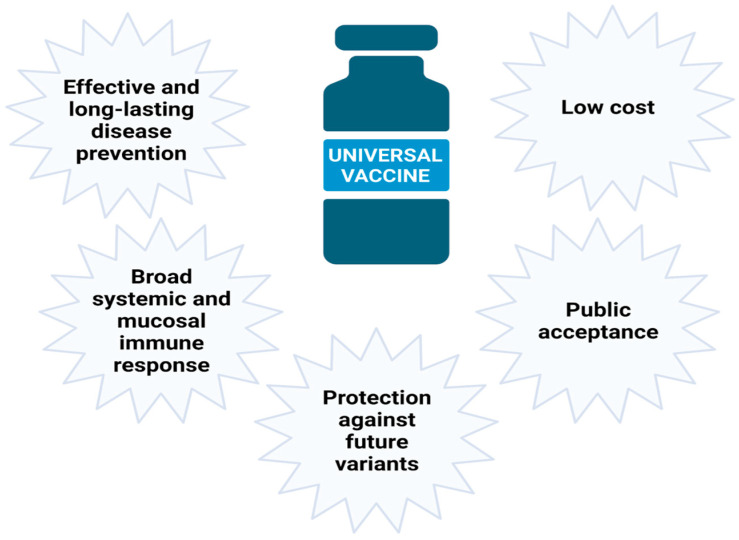
Key immunological, functional, and societal criteria for universal and pan-coronavirus vaccines. A universal coronavirus vaccine should integrate multiple design principles, including broad antigenic coverage targeting conserved viral regions, induction of both humoral and cellular immunity, and engagement of systemic and mucosal immune compartments. In addition to immunological efficacy, practical considerations such as durability of protection, limited dosing requirements, manufacturability, equitable global access, and public acceptance are critical for successful implementation. Together, these features define realistic and achievable benchmarks for the development of next-generation universal and pan-coronavirus vaccines.

Beyond defining the scope of protection, a universal or pan-coronavirus vaccine should strive to meet several desirable immunological, functional, and societal criteria that extend beyond the basic attributes of conventional vaccines (Figure 1). Rather than achieving absolute protection against all viral strains, a realistic and clinically meaningful goal is to elicit a broad and durable immune response capable of neutralizing or controlling multiple variants arising through antigenic drift. Such protection is most likely to be achieved by targeting conserved viral regions and engaging both humoral and cellular immune mechanisms to ensure cross-reactivity and resilience to viral evolution.

Equally important are considerations related to vaccine deployment and public acceptance. Innovative vaccine technologies, particularly those based on genetic platforms or novel delivery systems, often face increased public scrutiny, underscoring the need for robust safety data and transparent regulatory evaluation. A key advantage of broadly protective vaccines lies in their potential for extensive pre-epidemic testing and early regulatory approval. Unlike variant-specific vaccines that must be rapidly designed, tested, and authorized during an active outbreak, a universal vaccine could be developed, evaluated, and licensed well in advance of future epidemics. This approach would allow comprehensive preclinical and clinical studies under standard regulatory conditions, ensuring robust evidence for safety and efficacy before deployment.

Early approval would also facilitate large-scale manufacturing and stockpiling, enabling rapid deployment when a novel coronavirus emerges. Importantly, validation through conventional regulatory pathways, rather than accelerated emergency procedures, may strengthen public trust and improve vaccination uptake. Ideally, a universal vaccine would confer robust protection with a limited number of doses, induce both systemic and mucosal immunity, and maintain long-term effectiveness across diverse populations. Affordability and equitable global access, particularly in low- and middle-income countries, remain essential prerequisites for achieving meaningful public health impact [14].

Progress toward this goal further requires a detailed understanding of correlates of protection following natural infection and vaccination. Analyses of immune durability, tissue localization (systemic versus mucosal), and functional quality of immune responses are critical for informing immunogen design and vaccination strategies. Human challenge studies using endemic coronaviruses, such as HCoV-OC43, may provide valuable mechanistic insights into protective immunity and optimal routes of administration [14]. Supporting the feasibility of broad coronavirus protection, preclinical evidence has demonstrated that a SARS-CoV vaccine developed during the 2002–2004 outbreak elicited cross-reactive immune responses against SARS-CoV-2 in mice, highlighting the potential of conserved antigenic targets to mediate protection across related coronaviruses [15].

Taken together, these considerations underscore both the necessity and the feasibility of developing universal coronavirus vaccines. While current SARS-CoV-2 vaccines have substantially reduced severe disease and mortality, their effectiveness against infection declines as new variants emerge, necessitating repeated reformulation and administration [4,5,6,7,8,9,16,17,18,19]. A universal vaccine capable of maintaining protection despite continuous viral evolution would therefore represent a transformative advance in global pandemic preparedness, paralleling ongoing efforts toward universal influenza vaccines [20,21].

### 2.3. Cost-Effectiveness and Public Health Benefits of a Universal Coronavirus Vaccine

A universal (pan-X) vaccine could reduce disease severity, accelerate viral clearance, and decrease morbidity and mortality during the early stages of a pandemic, before a strain-specific vaccine becomes available [20]. A universal vaccine would also ensure that the population obtains at least some degree of protection as quickly as possible. It has further been suggested that immunization with such a vaccine could reduce viral transmission from vaccinated but infected animals, thereby decreasing the overall size and geographic spread of future epidemics. In addition, deploying a universal, “on-the-shelf” pandemic-preparedness vaccine could yield significant economic benefits. To evaluate these potential impacts, a computational simulation model was developed to represent the spread and consequences of different coronaviruses in the United States and to assess various types of universal and strain-specific coronavirus vaccines. The model incorporated factors such as hospitalizations, deaths, quality-adjusted life years (QALYs) lost, productivity losses, direct medical costs, and total societal costs. It would also provide valuable time to better understand the pathogen and prepare highly effective, variant-specific vaccines capable of completely suppressing the pandemic [22]. Although a universal vaccine may not provide the same level of protection as a strain-specific vaccine, the findings indicated that it would be cost-effective, and even cost-saving, as a stand-alone intervention, even when its efficacy was as low as 10% and coverage reached only 10% of the U.S. population. Moreover, every 1% increase in efficacy between 10% and 50% was estimated to avert an additional 395,000 infections and save approximately $1.0 billion in total societal costs, including $45.3 million in productivity losses and $1.1 billion in direct medical costs [22]. The model also assumed that, even if a strain-specific coronavirus vaccine were to become available later, a universal vaccine with relatively modest efficacy would remain cost-saving as long as the development, testing, and rollout of the strain-specific vaccine required at least 2–3 months [22]. Overall, these results provide strong support for the continued development of a universal coronavirus vaccine, underscoring not only its potential public health benefits but also its economic justification.

## 3. Rational Antigen Design Strategies for Universal and Multi-Epitope Vaccines

### 3.1. Epitope-Centered Framework for Universal Vaccine Design

The development of a universal SARS-CoV-2 vaccine is fundamentally constrained by immunological principles governing antigen recognition, such that—irrespective of the vaccine platform or route of administration—the achievable breadth of protection is ultimately defined at the level of antigen and epitope selection.

Conserved viral epitopes that are functionally constrained and evolutionarily stable constitute the primary determinants of cross-variant and cross-species protection. Accordingly, technological advances—including nanoparticle platforms, adjuvants, and alternative delivery routes—cannot compensate for suboptimal epitope selection, but instead function to enhance the immunogenic visibility and functional relevance of appropriately chosen epitopes.

Therefore, antigen and epitope design should be regarded as the first and non-substitutable step in universal vaccine development, providing the immunological foundation upon which downstream formulation and delivery strategies are built. Within this epitope-centered framework, the following sections examine how these principles are implemented through rational antigen design strategies.

Building on this framework, vaccine development necessarily begins with the discovery and identification of protective antigens, which remain central determinants of universal vaccine design. Despite major advances in vaccine delivery systems and adjuvant technologies, the careful selection and rational engineering of vaccine antigens continue to shape the breadth, durability, and effectiveness of immune protection. This principle is especially evident in universal and multi-epitope vaccine strategies, which aim to overcome pathogen diversity, antigenic variation, and immune escape by targeting conserved and functionally constrained antigenic regions.

### 3.2. Conserved Antigenic Regions of SARS-CoV-2 as Promising Targets for Universal Vaccine Development

From the perspective of universal vaccine development, conserved antigenic regions represent an immunological prerequisite rather than a complementary target, as they define the upper limit of achievable cross-variant protection. Among these, the trimeric spike glycoprotein plays a central role in viral entry by mediating attachment to the host angiotensin-converting enzyme 2 (ACE2) receptor and subsequent membrane fusion, making it the primary target of neutralizing antibodies and current vaccine strategies. Structural studies have revealed that the S protein contains both highly immunogenic but variable regions, and more conserved epitopes that are functionally constrained and less tolerant to mutation. From a universality standpoint, these functionally constrained epitopes represent indispensable targets, as their limited mutational tolerance defines the achievable breadth of vaccine-induced protection. In contrast, the N protein, while highly conserved and abundantly expressed, is internal and primarily elicits T-cell responses rather than neutralizing antibodies, whereas the E and M proteins contribute to virion assembly and stability but are weakly immunogenic. Importantly, antibody responses directed against certain spike epitopes may not only vary in neutralizing potency but can also contribute to the antibody-dependent enhancement of infection under specific conditions, underscoring the need for careful antigen selection and epitope-focused design. Together, the structural and functional organization of SARS-CoV-2 highlights both opportunities and challenges for universal vaccine development, emphasizing the need to balance broad cross-reactivity, durable protection, and immunological safety through rational virus and antigen design.

**Figure 2:**
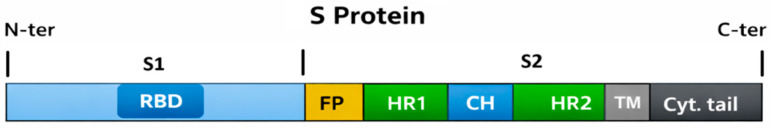
The S protein is a trimeric class I fusion glycoprotein composed of the variable S1 subunit, containing the RBD, and the more conserved S2 subunit. The S2 subunit includes the FP, CH, HR1 and HR2, transmembrane domain (TM), and cytoplasmic tail, which collectively mediate membrane fusion. Due to strong functional constraints, these S2 elements exhibit limited sequence variability across SARS-CoV-2 variants and related sarbecoviruses, underscoring their potential as targets for broadly protective coronavirus vaccines.

The S protein (Figure 2) consists of two functional subunits, S1 and S2. The S1 subunit mediates receptor recognition and contains the receptor-binding domain (RBD), while the S2 subunit facilitates membrane fusion and viral entry into host cells. The RBD within the S1 subunit interacts with the human ACE2 receptor, enabling viral attachment and entry. Owing to this crucial role in infection and its high immunogenicity, the RBD has been identified as the most promising target for eliciting protective immune responses, and the S protein remains the principal antigen used in most currently approved vaccine formulations [23,24,25,26]. However, despite its immunological importance, the RBD also represents the most mutation-prone region of the SARS-CoV-2 genome. This high mutational frequency is largely driven by the intrinsic variability of the S1 subunit, which harbors the RBD and is subject to strong selective pressure from host immune responses. As a consequence, the RBD has accumulated substitutions at numerous positions across globally circulating lineages, facilitating the emergence of SARS-CoV-2 variants with altered transmissibility, antigenicity, and immune evasion capacity. Early genomic surveillance revealed rapid diversification of the RBD, with multiple amino acid positions exhibiting recurrent variation across independent viral lineages [27], highlighting its comparatively low genetic stability. Consequently, vaccines that rely exclusively on the RBD as an immunogen are particularly susceptible to reductions in effectiveness as antigenically divergent variants continue to arise [28,29,30,31].

An alternative and promising strategy for universal coronavirus vaccine development involves targeting conserved regions of the S protein that are subject to strong functional constraints and therefore exhibit limited sequence variability. Among these regions, the S2 subunit is considerably more conserved across SARS-CoV-2 variants than the immunodominant S1 subunit, making it an attractive target for broadly protective vaccine design [32]. Structurally, the S2 subunit comprises several functionally essential elements involved in membrane fusion, including the fusion peptide (FP), the central helix (CH), and the heptad repeat regions (HR1 and HR2), which together form the six-helix bundle required for viral–host membrane fusion and entry. Within class I viral fusion proteins, the fusion peptide plays a critical role by inserting into the host cell membrane and initiating the fusion process. Due to this indispensable function, FP sequences are highly conserved within virus families characterized by rapid antigenic evolution, including coronaviruses, influenza viruses, and human immunodeficiency viruses [33,34]. This high degree of amino acid conservation reflects strong evolutionary pressure to maintain membrane fusion competence and limits the capacity of these regions to tolerate mutations. Consequently, the FP and associated HR1–HR2 motifs within the S2 subunit represent promising targets for the design of broadly protective or universal vaccines capable of eliciting cross-reactive immune responses.

Importantly, conserved elements within the S2 subunit are shared not only among SARS-CoV-2 variants but also across other sarbecoviruses, supporting the feasibility of cross-protective immunity against both currently circulating and future zoonotic coronaviruses [35]. Experimental evidence further supports this concept, as S2-based vaccine approaches have been shown to induce broadly neutralizing antibodies in vitro and in animal models. Studies by Ng et al. and Halfmann et al. demonstrated that immunization strategies targeting conserved S2 epitopes elicited neutralizing immunity against diverse α- and β-coronaviruses, including multiple Omicron subvariants and related animal coronaviruses [36,37].

Nevertheless, the rational design of universal vaccine antigens based on conserved SARS-CoV-2 proteins must also account for potential cross-reactivity with host self-antigens. Reports describing autoimmune manifestations following coronavirus infection or vaccination suggest that partial molecular mimicry between viral and human proteins may occur [38,39]. Therefore, comprehensive structural and immunological mapping of conserved S2 epitopes, particularly with respect to their potential overlap with human autoantigens or commensal microbiome-derived proteins, is essential to ensure vaccine safety and minimize unintended immune responses.

**Figure 3:**
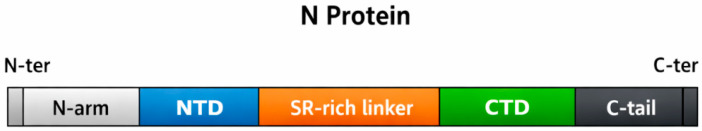
Schematic representation of SARS-CoV-2 N protein domain architecture. The protein comprises two structurally conserved domains: the N-terminal RNA-binding domain (NTD) and the C-terminal dimerization domain (CTD), connected by an intrinsically disordered linker region enriched in serine/arginine (SR-rich) residues. Additional disordered segments at the N-terminus and C-terminus (N-arm and C-tail) flank these domains and contribute to the dynamic flexibility and multivalent RNA interactions of the full-length protein.

Another highly conserved structural protein of SARS-CoV-2 is N protein (Figure 3), which plays a central role in viral RNA replication, transcription, and nucleocapsid assembly [40]. Structurally, the N protein consists of two well-defined and conserved domains: the N-terminal RNA-binding domain (NTD) and the C-terminal dimerization domain (CTD), which are connected by an intrinsically disordered, serine/arginine-rich linker region. These domains are essential for viral genome packaging, ribonucleoprotein complex formation, and interaction with the replication–transcription machinery, imposing strong functional constraints on sequence variability [40]. Consistent with these structural and functional requirements, the N protein exhibits a much lower mutation rate compared with the spike protein [41,42]. Bioinformatic and comparative sequence analyses have demonstrated a high degree of sequence conservation within the NTD and CTD across SARS-CoV-2 variants and related coronaviruses, highlighting their evolutionary stability and essential role in the viral life cycle [42]. Moreover, strong immunogenicity of the N protein has been observed in previous coronavirus infections, with N-specific immune responses contributing to T-cell activation and long-lasting immune memory [43]. Together, these features make the nucleocapsid protein an attractive target for the development of broadly protective and potentially universal coronavirus vaccines. Viewed in this broader context, the coexistence of highly immunogenic yet variable spike-derived targets and more conserved non-spike antigens highlights the need to systematically compare complementary antigen design strategies for achieving breadth, durability, and evolutionary resilience in universal coronavirus vaccines.

Current antigen-targeting strategies for universal SARS-CoV-2 vaccines can be broadly grouped into three complementary categories: (i) RBD-focused breadth strategies, including multivalent, mosaic, and chimeric designs that expand antibody coverage across circulating variants; (ii) conserved epitope-based approaches targeting structurally constrained regions of the spike protein, such as the S2 fusion peptide, stem-helix, and HR1–HR2 motifs, which offer enhanced evolutionary stability but reduced intrinsic immunogenicity; and (iii) non-spike antigen strategies, particularly those leveraging conserved epitopes from internal proteins such as nucleocapsid or ORF1ab-derived T-cell targets, which prioritize variant-resistant cellular immunity. Each of these approaches addresses a distinct aspect of viral evolution and immune escape, and none alone is likely to achieve complete universality, underscoring the need for integrated antigen design strategies.

Several promising strategies are currently being explored for the design of universal SARS-CoV-2 vaccine formulations, many of which build upon the identification of conserved viral epitopes with limited sequence variability. While such conserved regions, particularly within the S2 subunit of the spike protein or the nucleocapsid protein, offer a clear advantage in terms of evolutionary stability and cross-variant coverage, they often exhibit low immunogenicity when administered as isolated, monomeric antigens. In contrast to the immunodominant but highly variable RBD, conserved epitopes are frequently subdominant and poorly accessible to the immune system, resulting in weak antibody responses and limited protective efficacy. This phenomenon is best understood in the context of immunodominance. This limited immunogenicity is largely a consequence of immunodominance, whereby highly exposed but variable regions such as the RBD preferentially recruit B-cell responses, effectively masking conserved subdominant epitopes from immune recognition. Immunofocusing strategies, through antigen engineering, epitope masking, or multivalent presentation, aim to overcome this hierarchy by selectively amplifying immune responses directed toward conserved targets. To overcome these limitations, advanced antigen-delivery and presentation platforms are being actively developed. Among these, nanoparticle-based vaccine systems have emerged as particularly promising, as they enable multivalent and repetitive display of conserved epitopes in a highly ordered, virus-like geometry that enhances B-cell receptor cross-linking and germinal center formation. By improving antigen density, orientation, and stability, such platforms can substantially amplify immune recognition and promote the induction of broadly reactive and durable immune responses. Consequently, the combination of structurally conserved epitopes with rationally engineered presentation platforms represents a key strategic direction for the development of next-generation universal coronavirus vaccines.

### 3.3. In Silico Epitope Vaccine Design Targeting Conserved T-Cell Epitopes in Universal SARS-CoV-2 Vaccines

The increasing availability of genomic, structural, and immunological data has enabled the widespread application of in silico approaches to vaccine design. In the context of universal vaccine development, conserved T-cell epitopes define an independent and mechanistically distinct axis of universality, as they are largely resilient to antigenic drift affecting dominant antibody targets. Within this framework, T-cell-centered vaccine strategies do not rely on neutralization of viral entry but instead target conserved intracellular viral processes that are far less permissive to antigenic drift. Reverse vaccinology and structural vaccinology facilitate the systematic identification of conserved CD4^+^ and CD8^+^ T-cell epitopes with high immunogenic and protective potential, while excluding antigenic regions unlikely to confer durable immunity.

T cells recognize short peptide fragments presented by highly polymorphic Human Leukocyte Antigen (HLA; MHC) molecules, which are frequently derived from internal or functionally constrained pathogen proteins. Because mutations in these regions often compromise pathogen fitness, such epitopes tend to be evolutionarily conserved. As a consequence, T-cell epitopes frequently remain stable across viral variants and, in some cases, across related species, enabling cross-reactive and variant-resistant cellular immunity. These characteristics make conserved T-cell targets particularly attractive for the development of universal vaccines [44].

This strategy is especially relevant in the context of SARS-CoV-2, where rapid viral evolution has repeatedly reduced the effectiveness of antibody-based protection against infection. In contrast, numerous CD4^+^ and CD8^+^ T-cell epitopes derived from both structural and non-structural SARS-CoV-2 proteins remain highly conserved across variants, including Omicron lineages, allowing broad cellular recognition despite extensive mutations in dominant neutralizing antibody targets [45,46]. The persistence of these conserved epitopes provides a strong conceptual rationale for T-cell-focused universal vaccine designs aimed at achieving variant-independent protection [47]. Consistent with this notion, naturally induced SARS-CoV-2-specific T-cell responses exhibit substantial cross-recognition of variant epitopes, supporting the durability of cellular immunity across divergent viral lineages [46]. Viewed in the context of universal vaccine design, these observations position T-cell-focused strategies not as a replacement for antibody-mediated protection, but as a complementary and mechanistically distinct pathway to achieving breadth and resilience against viral evolution. Accordingly, effective universal vaccine design is increasingly viewed not as the optimization of a single antigen class, but as the rational integration of complementary B-cell- and T-cell-directed strategies capable of withstanding continuous viral evolution.

Despite these advantages, universal vaccine strategies based primarily on T-cell immunity face notable challenges. Extensive HLA polymorphism complicates epitope selection and necessitates multi-epitope formulations to ensure broad population coverage. Moreover, T-cell-based vaccines are unlikely to confer sterilizing immunity, as cellular responses act predominantly after infection has been established. Additional limitations include immunodominance effects, variability in antigen processing and presentation, and the lack of standardized correlates of protection.

These immunological principles have directly informed the development of epitope-focused and multi-epitope vaccine strategies, several of which have provided experimental evidence supporting the feasibility of conserved antigen targeting for universal SARS-CoV-2 vaccine development. Recent studies have demonstrated that multi-epitope vaccines composed of highly conserved human B-cell, CD4^+^, and CD8^+^ T-cell epitopes derived from multiple SARS-CoV-2 proteins can induce balanced humoral and cellular immune responses and confer robust cross-protection against severe disease and mortality caused by multiple variants of concern [48]. Complementary evidence from vaccine designs exclusively based on conserved CD4^+^ and CD8^+^ T-cell epitopes maintained from Alpha through Omicron variants further supports the capacity of T-cell-focused approaches to elicit strong and broadly reactive cellular immunity [49].

Collectively, the identification of conserved antigenic regions (Section 3.2) and the rational, bioinformatics-guided selection of B- and T-cell epitopes (Section 3.3) provide a strong conceptual foundation for universal SARS-CoV-2 vaccine design. These findings indicate that epitope-focused and multi-epitope antigen engineering represents a viable and promising pathway toward the development of vaccines capable of inducing durable, variant-independent immune protection. However, conserved epitopes, particularly those derived from functionally constrained regions such as the S2 subunit of spike or internal proteins like nucleocapsid, are often subdominant and poorly immunogenic when administered as isolated or monomeric antigens. Consequently, their effective incorporation into vaccine formulations requires advanced strategies for antigen presentation and immune focusing. To overcome these limitations, next-generation vaccine platforms capable of enhancing epitope density, structural stability, and multivalent display have been developed. Among these, nanoparticle-based delivery systems as well as hybrid and multivalent antigen designs have emerged as particularly promising approaches for amplifying immune recognition and promoting broad, durable, and cross-variant protection, as discussed in the following sections. Importantly, B-cell- and T-cell-directed vaccine strategies should not be viewed as competing approaches to universality, but rather as mechanistically complementary pathways through which conserved epitopes can confer protection against antigenically diverse coronaviruses [50]

### 3.4. Nanoparticle-Based Vaccine Platforms for Broad-Spectrum and Universal Coronavirus Immunization

Nanoparticle-based vaccine platforms do not generate immunological breadth per se; rather, they function as structural and immunological amplifiers of epitope-focused vaccine design, enhancing antigen density, stability, immunodominance, and immunofocusing toward conserved or heterologous epitopes. To address the limited immunogenicity of conserved SARS-CoV-2 epitopes and to enable their effective presentation to the immune system, nanoparticle-based vaccine platforms have emerged as a distinct and increasingly important strategy for achieving broad and durable immune responses. These platforms function as antigen carriers that enhance epitope density, stability, and multivalent display, thereby improving immune recognition. It has been demonstrated that nanoparticle-based vaccines are taken up more efficiently by antigen-presenting dendritic cells [51,52], and that multivalent antigen presentation enhances immune cell activation [53,54]. Nanoparticles are defined as nanoscale structures that typically mimic the physical properties of natural viruses, a feature that enables the induction of high titers of neutralizing antibodies and results in a broader immune response. Two major categories of nanoparticle use can be distinguished. The first includes particles encapsulating antigens or nucleic acids, while the second comprises particles designed to present vaccine antigens on their surface. These groups serve different purposes: nanoparticles carrying internal antigens protect and control antigen release upon administration, whereas surface-displaying nanoparticles aim to stimulate antigen-presenting cells and induce strong immunogenicity to the exposed protein [55].

Zhang et al. developed a nanoparticle-based vaccine displaying six receptor-binding domains (RBDs) derived from key Omicron and Delta SARS-CoV-2 sublineages, along with several highly conserved T-cell epitopes shared among sarbecoviruses (pan-sarbecovirus-aimed). Neutralization assays demonstrated inhibition of all tested pseudotyped variants, including major pandemic strains such as BA.2, BA.5, BA.2.75, BF.7, BQ.1.1, BQ.1, and XBB (pan-variant). Notably, the vaccine induced broad mucosal immunity with cross-protective activity even in the absence of an adjuvant, marking a significant step toward the development of a truly universal formulation [56].

Similarly, Qin et al. constructed a heterologous mRNA vaccine (4N4T-HRBD dodecamer) encoding a lipid nanoparticle (LNP)-delivered RBD multimer that incorporated tandem RBDs from the Delta, Beta, Gamma, and wild-type SARS-CoV-2 strains (pan-variant). This vaccine elicited a five-fold higher Delta RBD-binding antibody titer compared with those induced by the S protein, RBD monomer, or tetramer. Moreover, it generated potent neutralizing immunity against multiple variants, including Alpha, Delta, Beta, Gamma, and Omicron (pan-variant)**.** Immunized mice developed strong and durable immune responses, characterized by the activation of both CD4^+^ and CD8^+^ T cells. These findings suggest that RBD-based heterologous dodecamer antigens, when combined with LNP delivery, represent a promising strategy for the development of next-generation broad-spectrum mRNA vaccines [57].

In a preclinical study, Qin et al. evaluated an mRNA vaccine platform formulated in LNPs and encoding either a prefusion-stabilized Omicron Spike protein (OVS) (pan-variant; variant-adapted), a conserved multi-T-cell epitope cassette (MTE) (pan-variant; T-cell-biased), or a combination of both (pan-variant; hybrid B/T strategy). BALB/c and K18-hACE2 mice were immunized and subsequently challenged with either mouse-adapted SARS-CoV-2 MA30 or the Delta variant. Immunization elicited robust antigen-specific responses: the OVS construct induced high titers of binding and neutralizing antibodies, whereas the MTE construct generated strong CD4^+^ and CD8^+^ T-cell responses in the absence of detectable neutralizing antibodies. Notably, vaccination with MTE alone conferred substantial protection from lethal challenge, significantly reducing viral loads and lung pathology, while the combined OVS/MTE formulation provided the most comprehensive protection, achieving full survival and near-sterilizing immunity. These findings demonstrate that T-cell-directed vaccines can mitigate severe disease independently of neutralizing antibodies and that combining conserved T-cell epitopes with variant-adapted Spike antigens may enhance both the breadth and durability of protection [58].

Cohen et al. investigated mosaic-8b, a protein-based nanoparticle displaying eight diverse sarbecovirus RBDs, as a heterologous booster for animals previously immunized with various COVID-19 vaccines (pan-sarbecovirus). Designed to focus immune responses on conserved RBD epitopes, the nanoparticle presented non-identical RBDs in a randomized arrangement. Mosaic-8b elicited markedly broader binding and neutralizing responses compared with homotypic SARS-CoV-2 RBD nanoparticles or additional doses of mRNA, DNA, or adeno-viral vaccines. Molecular analyses revealed that mosaic-8b effectively recalled cross-reactive antibodies induced by prior vaccination while simultaneously generating antibodies against variant RBDs represented on the mosaic particle. These effects were not observed with homotypic boosters, suggesting that mosaic-8b may overcome immune imprinting and broaden epitope coverage. The authors further proposed that inclusion of clade-3 sarbecovirus RBDs or computationally optimized designs could further expand the breadth of immune protection [59].

Among nanoparticles-based platforms, ferritin-based nanoparticles have emerged as a particularly promising technology. Ferritin is an exceptionally stable protein complex that self-assembles into a highly ordered nanocage composed of twenty-four subunits, enabling precise and repetitive antigen presentation. This scaffold has been engineered to display viral glycoproteins from pathogens including influenza virus, RSV, HIV, Epstein–Barr virus, and betacoronaviruses. Increasing evidence suggests that multivalent ferritin nanoparticles displaying diverse antigens generate stronger and broader neutralizing activity than monovalent immunogens. Their ordered antigen presentation enhances B-cell receptor cross-linking, directs antibody responses toward conserved epitopes, and promotes robust T-cell activation and prolonged germinal center activity.

Martinez et al. evaluated two ferritin-based vaccine candidates—Spike-Ferritin Nanoparticle (SpFN) and Receptor-Binding Domain Ferritin Nanoparticle (RFN) derived from the ancestral WA-1 strain. Horses immunized with these candidates developed high-titer, broadly reactive hyperimmune sera. Passive transfer of purified equine IgG partially protected K18-hACE2 mice challenged with Omicron XBB.1.5 (pan-variant), reducing viral loads and disease severity. The authors highlight the potential of ferritin-based hyperimmunization as a scalable source of broad polyclonal therapeutics, though further evaluation in humans is needed [60]. Weidenbacher et al. developed DCFHP, a ferritin nanoparticle presenting an engineered HexaPro ∆C70 spike trimer optimized for stability, manufacturability, and immunogenicity. Formulated with alum, DCFHP induced strong neutralizing antibody responses against Wuhan-1, multiple Omicron variants, and SARS-CoV-1 (pan-sarbecovirus) in rhesus macaques, with responses persisting for over 250 days and boosted significantly after one year. A balanced Th1/Th2 CD4^+^ T-cell response was also observed. The authors emphasize the platform’s suitability for global deployment [61]. The recombinant ferritin nanoparticle SARS-CoV-2 vaccine SpFN, adjuvanted with the Army Liposomal Formulation containing monophosphoryl lipid A and QS-21 (ALFQ), has demonstrated potent protective efficacy in preclinical models [62,63], and a phase 1 clinical trial confirmed its safety, tolerability, and broad immunogenicity [64].

Current studies aim to optimize ferritin nanoparticles for heterologous antigen presentation to improve cross-clade sarbecovirus neutralization, an important step toward the development of a universal pan-betacoronavirus vaccine [65]. Ferritin-based nanoparticle vaccines thus represent a major advancement toward “all-in-one” universal platforms capable of integrating diverse sarbecovirus antigens into a single formulation. However, substantial progress is still needed before this goal can be fully realized. Highly conserved antigenic targets, both in sequence and structure, are expected to form the foundation of next-generation mosaic pan-sarbecovirus vaccine candidates.

### 3.5. Hybrid and Multivalent Antigen Strategies to Achieve Cross-Variant Immunity Against SARS-CoV-2

In the development of universal vaccines, several strategies focus on designing recombinant hybrid antigens composed of fused fragments derived from native SARS-CoV-2 proteins. This approach aims to generate immunogens that combine conserved viral regions with variable domains from multiple variants, thereby broadening immune coverage and enhancing cross-protective potential.

One example of this strategy involves the creation of subunit vaccine candidates based on a conserved spike (S) protein backbone, each encoding a distinct version of the receptor-binding domain (RBD) derived from different variants or the ancestral strain. Among these constructs, the formulation incorporating the Delta variant RBD elicited exceptionally strong and broadly neutralizing antibody responses, conferring complete protection in mice against all tested SARS-CoV-2 variants, including Alpha, Beta, Gamma, Delta, and multiple Omicron subvariants (BA.1, BA.2, BA.2.75, BA.4.6, and BA.5), as well as the wild-type strain [66]. These findings identify the Delta RBD-based subunit vaccine as a promising candidate for universal SARS-CoV-2 vaccine development. Further studies in additional animal models, including non-human primates (NHPs), are required to evaluate the durability of induced immune responses and long-term protection [62]. Overall, these results represent a promising strategy toward achieving broad-spectrum immunity against current and future coronavirus variants.

The use of chimeric immunogens is an increasingly explored approach in the development of vaccines capable of inducing broad immune responses against emerging SARS-CoV-2 variants. This strategy involves combining key immunogenic components from different variants into a single antigen to elicit cross-protective antibodies. Chimeric designs can be applied to individual protein domains (e.g., RBD) as well as full-length proteins (e.g., S protein), and their modular nature makes them well suited for rapid immunogen updates, which is a critical advantage for responding to newly arising epidemics. Appelberg et al. developed the OC-2.4 DNA vaccine, which encodes three unique RBD loop regions corresponding to WH1, Alpha, and Beta variants, as well as the M and N proteins. In vitro analyses demonstrated that this antigen induced high levels of antibodies capable of neutralizing the WH1, Beta, Delta, and Omicron variants. T-cell activation contributed substantially to protection in mice by limiting viral replication [67]. Based on earlier work, Xu et al. designed a Delta–Omicron heterodimer vaccine that induced broad neutralizing immunity against Alpha, Beta, Delta, and Omicron strains. Comparative analyses showed that the Delta–Omicron heterodimer generated a much broader and more effective response than a Beta RBD homodimer [68]. Zhang et al. engineered the DBA2BA5 RBD heterotrimer, composed of RBDs from the Delta, BA.2, and BA.5 variants. This construct induced broad neutralizing immunity against SARS-CoV-2 variants, including XBB, BQ.1.1, and BF.7. Challenge studies confirmed high protective efficacy correlating with strong neutralizing antibody titers [69]. Han et al. developed an mRNA vaccine expressing hetero-chimeric RBD dimers from Delta and Omicron variants. The vaccine elicited broad neutralization against Alpha, Beta, Delta, and Omicron pseudoviruses, and induced a strong Th1-skewed cytokine response without excessive Th2 activation. The chimeric Delta–Omicron immunogen enhanced cross-variant neutralization and demonstrated the feasibility of rapid antigen updates within existing mRNA platforms [70]. Brinkkemper et al. engineered prefusion-stabilized S glycoproteins representing four distinct sarbecovirus clades (1a, 1b, 2, and 3), covalently coupled to influenza-derived virosomes. Vaccines were prepared either as a cocktail of monovalent virosomes or as a mosaic virosome displaying all four S proteins simultaneously. Both formats elicited broad binding responses, with multivalent virosomes generating more balanced and wider reactivity across sarbecoviruses. Neutralization assays confirmed heterologous activity, including against SARS-CoV, WIV1, Pangolin-CoV, and partial neutralization of SARS-CoV-2. The extended 6-week dosing interval markedly enhanced both titers and breadth. These findings support the concept that simultaneous presentation of antigenically diverse spikes can preferentially recruit B-cells targeting conserved epitopes, enabling the emergence of pan-sarbecovirus antibodies [71].

Across current strategies, three major groups of universal vaccine candidates can be distinguished. The first and largest group employs highly immunogenic viral proteins or domains (most commonly the S protein and RBD) from circulating SARS-CoV-2 variants. Many studies demonstrate that combining antigens from multiple variants elicits broader protection, potentially effective even against future strains. The second group focuses on viral proteins or domains with low mutational rates, such as the S2 subunit or the conserved structural proteins M, N, and E. Although less immunogenic, these targets may become more feasible through improved adjuvant technologies that enhance antigen presentation and immune activation. The third group integrates both strategies, combining highly immunogenic (but mutable) antigens with conserved domains to achieve strong and broad protection simultaneously. This hybrid approach appears particularly promising for future universal vaccine design.

## 4. Adjuvants: Fundamental Tools for Strengthening Immune Response

Adjuvants cannot substitute for rational antigen and epitope selection; however, they are essential for rendering conserved and often subdominant epitopes immunologically visible and functionally relevant. Subunit vaccines are inherently safe, primarily because they do not contain whole pathogens but only defined antigens. When designing a universal vaccine, it seems particularly advantageous to use antigens based on highly conserved regions of the virus. These regions, however, are typically weakly immunogenic and often unable to induce a sufficiently robust immune response. Enhancing the potential of such vaccines is therefore possible through the use of appropriate adjuvants. Adjuvants are immunostimulatory components of vaccine formulations that optimize antigen distribution and support the activation of immune cells. Many of the adjuvants currently used fulfill both of these functions. Developing an optimal antigen–adjuvant formulation may allow precise modulation of the immune response, resulting in a broad spectrum of neutralizing activity—an essential feature when combating pathogens with high antigenic diversity [72]. Among the most widely used compounds in licensed vaccines, including those targeting SARS-CoV-2, are aluminum salts [73]. Alum is a component of many vaccines currently in use against pathogens such as hepatitis A and B viruses, human papillomavirus, *Haemophilus influenzae* type B, or *Clostridium tetani* [74]. This adjuvant has been used for nearly 100 years, and until 2009 it remained the only adjuvant approved for use in the United States. Alum effectively supports humoral immunity by inducing a Th2-type response. Despite extensive knowledge about this adjuvant and its proven ability to stimulate humoral immunity, aluminum compounds also have several limitations. The most significant drawback is the weak induction of cellular immune responses, which makes alum suboptimal for vaccines targeting intracellular pathogens [75]. Additionally, vaccines containing aluminum adjuvants cannot be frozen or lyophilized, complicating their storage and transport [76]. For these reasons, there is an urgent need to develop next-generation adjuvants capable of enhancing antigen immunogenicity in a targeted and safe manner. Ideally, such adjuvants should be broadly applicable to various antigens and support long-term stimulation of systemic, cellular, and mucosal immunity [77]. It would also be beneficial if they were compatible with different vaccine formulations while maintaining their effectiveness. In recent years, several new adjuvants have been approved or have reached late-stage clinical trials. These include Toll-like receptor agonists (e.g., MPL), saponin-based adjuvants (e.g., QS-21), and squalene emulsions (MF59 and AS03). These and other emerging immunostimulatory compounds may significantly accelerate the development of a universal vaccine capable of pandemic prevention and control. The combined properties of adjuvants can help overcome obstacles that hinder the creation of an effective universal vaccine.

The immunostimulatory capacity of adjuvants enables a reduction in antigen dosage and a decrease in the number of booster doses. This may help meet increased vaccine demand in the early phases of a pandemic, when shortages are likely. Another important advantage is improved vaccine efficacy, particularly relevant for elderly individuals and newborns whose immune systems are less capable of generating effective immunity. Through the use of adjuvants, it is also possible to guide the immune response toward protective pathways. This is especially critical in the case of coronaviruses, where vaccine-associated enhancement linked to an imbalanced Th2 response remains a concern. Achieving a balanced Th1/Th2 response may therefore substantially improve the safety of future vaccines [74]. A promising direction in adjuvant development involves antigen carriers with inherent immunostimulatory properties. Recent studies by Lai et al. demonstrate that selenium nanoparticles may serve as highly effective antigen carriers with adjuvant-like behavior. They can efficiently deliver antigens and modulate the immune response by stimulating rapid immune cell proliferation, accelerating cytokine production, and shaping immunity to better counteract infection. A selenium-based nanovaccine containing the SARS-CoV-2 RBD maintained high IgM titers and demonstrated a 27-fold increase in inhibitory activity 21 days after immunization compared with RBD administered using a standard aluminum-based adjuvant. These results suggest that selenium nanoparticles may be strong candidates for inclusion in universal vaccine constructs, as they can substantially enhance and prolong immune responses—an important feature for vaccines targeting SARS-CoV-2 and other viruses [78]. Next-generation adjuvants have the potential to improve vaccine delivery, activate dendritic cells, facilitate antigen presentation, and enhance immunological memory. Close collaboration across disciplines such as immunology, materials science, and biomedical engineering will be essential for their further development [74]. Recent clinical studies indicate that incorporating diverse novel immunostimulatory molecules into vaccine formulations is advisable for future human use. The availability of advanced adjuvants in various combinations will support the strategic development of effective and truly universal vaccines [72].

## 5. Mucosal Route of Administration as a Promising Approach in Universal Vaccine Design

The route of vaccine administration modulates the anatomical and immunological context in which epitopes are encountered by the immune system but does not alter the fundamental requirement for conserved antigenic targets. Another important aspect that should be considered when designing a universal vaccine is the route of its administration. Most licensed vaccines against infectious pathogens are administered subcutaneously or intramuscularly. These methods effectively induce systemic immune protection, which is sufficient for pathogens causing disseminated infections. However, this approach is suboptimal for inducing local immunity at the primary entry sites of SARS-CoV-2 and other respiratory pathogens, namely the mucosal surfaces of the upper respiratory tract [79]. Unlike viruses that typically cause systemic infections (e.g., rubella, varicella, or measles), SARS-CoV-2 predominantly infects mucosal epithelial cells, resulting in limited and transient engagement of the systemic immune system. Consequently, vaccine-induced protection is often incomplete and short-lived, contributing to reinfections and limiting the effectiveness of systemic vaccination.

Research on universal SARS-CoV-2 vaccines should therefore aim to deepen our understanding of the interplay between systemic and mucosal immune responses elicited by natural infection and vaccination. In particular, there is a critical need to identify the humoral and cellular immune components that are essential for inducing broad, durable protection against continuously evolving coronaviruses. For these reasons, interest in mucosal vaccine strategies has grown substantially, as immune responses elicited directly at the site of viral entry may prevent infection, reduce viral replication, and limit transmission. Mucosal surfaces, including nasal, pulmonary, and gastrointestinal tissues, constitute the largest interface between the host and the external environment [80] and are predominantly protected by secretory IgA (sIgA). In contrast, intramuscular and subcutaneous vaccines induce only weak or negligible sIgA responses. The hypothesis that upper respiratory tract mucosal immunity provides superior protection against infection and transmission remains a major driver of mucosal vaccine development.

Mucosal vaccines against SARS-CoV-2 have been extensively reviewed elsewhere [80,81]. Although the lack of widespread implementation limits direct assessment of their real-world effectiveness, important insights can be drawn from natural infection. Individuals who recover from COVID-19 develop high titers of IgA antibodies in the respiratory tract, as well as tissue-resident memory T and B cells in the lungs [81,82,83,84,85]. In contrast, such mucosal immune responses are absent or minimal in vaccinated individuals without prior infection [84,86,87]. Epidemiological data further indicate that prior infection can reduce viral transmission, with protection against Omicron subvariants comparable to that conferred by three doses of current vaccines [88]. Longitudinal analyses suggest that past infection provided approximately 55% protection against pre-Omicron variants after 1.5 years [89], and reinfected individuals exhibit lower viral titers, consistent with reduced transmissibility [90]. Collectively, these observations highlight immune mechanisms that could potentially be recapitulated through vaccination strategies targeting the upper respiratory mucosa.

In response to the global demand for improved SARS-CoV-2 vaccination strategies, intensive research efforts have accelerated the development of mucosal vaccines. Several intranasal or inhaled vaccines have now received regulatory authorization, representing a significant milestone in the field. These include Convidecia Air (CanSino Biologics Inc., Tianjin, People’s Republic of China), an adenoviral-vectored intranasal vaccine approved as a heterologous booster; iNCOVACC (BBV154) (Bharat Biotech International Limited, Hyderabad, Telangana, India), authorized for emergency use as both a primary series and booster; Gam-COVID-Vac nasal (Gamaleya Research Institute of Epidemiology and Microbiology, Moscow, Russian Federation), granted limited authorization; an intranasal recombinant protein vaccine developed by Beijing Wantai Biological Pharmacy Enterprise Co., Ltd. (Beijing, People’s Republic of China); and Razi Cov Pars (Razi Vaccine and Serum Research Institute, Karaj, Alborz Province, Islamic Republic of Iran), approved in a combined intramuscular–intranasal prime–boost regimen. Importantly, most of these approvals were issued under emergency or conditional frameworks, and robust real-world effectiveness data, particularly regarding transmission blocking, remain limited.

Despite their promise, mucosal vaccines face several well-recognized challenges that must be addressed before they can be widely adopted as components of universal vaccine strategies. A major limitation is the durability of mucosal immune responses, especially sIgA, which often wanes more rapidly than systemic IgG and may necessitate frequent boosting. Dose standardization and delivery efficiency represent additional challenges, as intranasal deposition can vary substantially between individuals. Safety considerations, including local reactogenicity, inflammation, and rare neurological adverse events, require careful antigen selection and formulation. Moreover, correlates of mucosal protection remain poorly defined, complicating the evaluation and comparison of candidate vaccines. Finally, manufacturing scalability and distribution pose nontrivial obstacles, particularly for live attenuated or complex vectored vaccines designed for intranasal delivery.

Distinguishing between currently authorized mucosal vaccines and next-generation pipeline candidates is therefore essential for accurately assessing the translational potential of this approach. While authorized products provide proof of feasibility and safety in humans, experimental platforms offer opportunities to systematically optimize breadth, durability, and cross-species protection.

In this context, Yuen et al. [91] recently described a prototype mucosal vaccine with pan-sarbecovirus potential, termed IBIS (Interferon Beta-Integrated SARS-CoV-2). This rationally attenuated SARS-CoV-2 variant lacks a functional envelope (E) protein and replaces ORF8 with an interferon-β-encoding cassette. The design aims to enhance safety while recapitulating the early interferon response that is typically delayed during coronavirus infection.

In mouse and hamster models, intranasal IBIS immunization induced robust humoral and cellular immune responses, including anti-RBD IgG, anti-nucleoprotein antibodies, polyfunctional CD8^+^ T cells, and strong activation of mucosal CD4^+^ T cells. Notably, IBIS conferred complete protection against infection and transmission of the ancestral SARS-CoV-2 strain, multiple variants of concern (Delta; Omicron BA.1, BA.2, BA.4.1, BA.5.2), and heterotypic SARS-CoV-1. In many cases, viral titers in the lungs and nasopharynx were undetectable, and transmission was fully blocked in hamsters. From the perspective of universal vaccine development, these findings provide compelling evidence that mucosal delivery of appropriately engineered viral vaccines can induce broad, multidimensional, and cross-species immunity. They further demonstrate that mucosal immunization strategies are uniquely positioned to activate the integrated humoral and cellular immune responses required for achieving truly pan-sarbecovirus protection.

To facilitate comparison across the antigen design and delivery strategies discussed in Section 3.3 and Section 3.4, together with adjuvant- and mucosal-based approaches described in Section 4 and Section 5, Table 1 summarizes representative vaccine platforms, breadth endpoints, developmental stages, and key limitations relevant to universal SARS-CoV-2 vaccine development.

## 6. Learning from Vaccine-Induced Immunity

In long-term efforts to develop a universal vaccine against SARS-CoV-2, it is important not to focus solely on designing novel antigens, vaccine formulations, or delivery platforms. Equally essential is the systematic exploitation of the extensive immunological data accumulated since the beginning of the pandemic from individuals vaccinated with existing vaccines who had not been previously infected, as this cohort enables the assessment of vaccine-induced immunity independent of infection-driven immune imprinting. Analyzing humoral and cellular immune responses in such individuals can provide critical insights into the breadth, durability, and cross-protective potential elicited by current vaccines. For example, a cohort study of individuals vaccinated with a formulation adapted to the BA.4/5 subvariant demonstrated that, in participants without prior SARS-CoV-2 infection, anti-RBD IgG concentrations and neutralizing antibody titers remained significantly elevated even nine months after vaccination, indicating durable and partially broad immune responses [92]. Such observations can help identify conserved epitopes capable of mediating cross-reactive immunity against divergent viral variants.

Zhao et al. [93] provided important insights into how heterologous, multi-dose COVID-19 vaccination can shape the human B-cell repertoire toward both potent and broadly neutralizing antibody responses. In a donor who received two doses of the BNT162b2 vaccine followed by three doses of the inactivated BBIBP-CorV vaccine, the authors observed unusually strong cross-neutralizing serum activity extending across SARS-CoV-2 variants and even to MERS-CoV. Using antigen-specific memory B-cell sorting coupled with a structure-guided computational screening pipeline, they identified monoclonal antibodies with remarkable breadth. Among these, PW5-570 exhibited ultrapotent neutralization of pre-Omicron SARS-CoV-2 strains through high-affinity binding to receptor-binding motif residues essential for ACE2 engagement. More importantly for universal vaccine design, PW5-5 and PW5-535 targeted highly conserved, cryptic epitopes on the inner RBD surface and the RBD–SD1 interface—regions minimally affected by contemporary viral evolution. These antibodies induced substantial conformational rearrangements of the spike, including trimer destabilization, consistent with broad sarbecovirus activity. In golden Syrian hamsters, PW5-570 conferred strong prophylactic protection against Omicron BA.1, while PW5-5 and PW5-535 demonstrated both prophylactic and therapeutic efficacy against SARS-CoV-2 XBB.1 and SARS-CoV. Together, these findings confirm that conserved epitopes targeted by broadly neutralizing antibodies remain functionally accessible during infection and vaccination, highlighting the potential of sequential immunization and rational immunogen design to steer antibody responses toward conserved sarbecovirus determinants.

Recent comparative analyses [94] of neutralizing antibody kinetics and vaccine effectiveness further underscore the value of mining immunological data from vaccine-only cohorts. Systematic evaluation of neutralization and effectiveness studies revealed that, although neutralizing antibody titers decline rapidly and are substantially reduced against antigenically shifted variants such as Omicron, protection against severe and fatal COVID-19 remains remarkably stable, even when neutralization approaches the limits of assay detection. This dissociation between humoral neutralization and clinical protection strongly suggests that vaccine-induced immunity engages additional, more conserved components of the antiviral response. Moreover, differences in waning dynamics across vaccine platforms and heterologous booster regimens indicate that specific immunization strategies may more effectively amplify durable and cross-reactive immune responses.

Collectively, these observations provide a mechanistic framework for translating vaccine-induced immunity into rational design principles for broad-spectrum vaccines. First, data from heterologous and sequential vaccination regimens demonstrate that the order, composition, and timing of immunization can actively steer immune responses toward conserved and otherwise subdominant epitopes, supporting earlier arguments for rational immunofocusing strategies discussed in Section 3.2 and Section 3.4. Such principles are particularly amenable to implementation using modular vaccine platforms, including nanoparticle-based and multivalent antigen designs, which allow controlled epitope presentation and sequential antigen exposure.

Second, the identification of broadly neutralizing antibodies targeting cryptic, structurally conserved epitopes, such as the inner RBD surface or interdomain interfaces, confirms that these regions are functionally accessible during infection and vaccination despite being immunologically subdominant. This finding reinforces the value of conserved epitope-targeting strategies outlined in Section 3.1 and Section 3.2 and highlights the importance of advanced antigen engineering approaches capable of exposing or stabilizing such epitopes.

Third, comparative analyses of vaccine effectiveness indicate that neutralizing antibody titers alone are insufficient correlates of durable protection, particularly against severe disease. Instead, long-term protection appears to depend on a combination of antibody breadth, functional antibody activities, and cellular immunity. This observation aligns with the emphasis placed earlier on incorporating conserved T-cell epitopes and on vaccine platforms that promote balanced humoral and cellular responses, including nanoparticle-based systems and appropriately adjuvanted subunit vaccines.

Fourth, differences in immune durability observed across vaccine platforms and booster strategies demonstrate that vaccine formulation and delivery context critically shape the quality and longevity of immune memory. These insights underscore the importance of integrating immunological readouts from vaccine-only cohorts into platform selection and adjuvant optimization, as discussed in Section 3.3 and Section 4.

Taken together, learning from vaccine-induced immunity in infection-naive individuals does not merely validate existing approaches but yields actionable design rules that can be directly translated into next-generation vaccine strategies. By systematically integrating these lessons with conserved antigen targeting, computational epitope design, and advanced delivery platforms, the field can move toward more predictive and mechanism-driven development of broadly protective and potentially universal coronavirus vaccines.

## 7. Discussion

From a conceptual and immunological standpoint, the development of a universal SARS-CoV-2 vaccine should be approached as a hierarchical design process rather than a platform-driven optimization problem. The identification of conserved, functionally constrained B-cell and T-cell epitopes represents the primary and non-substitutable determinant of universality, as these elements define the upper limit of achievable breadth and durability of protection. Rational antigen and epitope engineering should therefore precede and inform all downstream considerations, including vaccine formulation, adjuvant selection, and route of administration, which serve to amplify and shape epitope-driven immune responses rather than to generate breadth independently. The continued emergence of SARS-CoV-2 variants and sublineages with increased transmissibility and immune escape capacity challenges the effectiveness of existing vaccination approaches, underscoring the need for universal vaccine strategies despite the substantial technical challenges associated with their development. Our analysis of key factors, including antigen selection, adjuvant utilization, and routes of administration, highlights critical considerations for advancing vaccine design and improving overall efficacy. Rather than relying on a single solution, the available evidence suggests that vaccine “universality” is more likely to arise from the integration of complementary antigen design strategies, each engaging distinct components of the immune system and addressing different aspects of viral evolution and immune evasion. At the same time, navigating the complex interplay between antigen immunogenicity and host immune responses remains challenging and requires careful evaluation to ensure optimal and durable protection.

The COVID-19 pandemic has underscored the substantial threat posed by rapidly evolving respiratory viruses and the inherent limitations of strain-matched vaccination strategies. The emergence of highly transmissible SARS-CoV-2 variants, including Delta and Omicron, demonstrated that mutations within vaccine-targeted regions can reduce protection against infection and facilitate continued viral spread, even in vaccinated populations. While currently licensed vaccines remain highly effective at preventing severe disease and mortality, their reduced durability against infection highlights the need for more flexible immunization strategies that are resilient to ongoing viral evolution. These observations provide a strong biological and public health rationale for the development of universal and pan-coronavirus vaccines capable of maintaining protective efficacy across current and future variants.

Targeting conserved antigenic regions represents a central principle of universal vaccine design. Vaccines incorporating such regions can induce cross-reactive immune responses, although the underlying mechanisms differ substantially. Conserved B-cell epitopes primarily expand the breadth of antibody recognition, often at the expense of reduced neutralization potency, whereas conserved T-cell epitopes contribute to variant-resistant cellular immunity, which is particularly effective at limiting severe disease rather than preventing infection. In this context, cytotoxic CD8^+^ T cells play a critical role by eliminating virus-infected cells, thereby controlling viral replication and disease progression. Consequently, T-cell-mediated immunity is mechanistically distinct from, yet functionally complementary to, antibody-mediated protection.

Hybrid and multivalent antigen strategies exemplify this immunological complementarity. Chimeric antigens, which combine conserved and variable regions within a single immunogen, aim to redirect immune responses toward subdominant but conserved epitopes and may reshape established immunodominance hierarchies. In contrast, multivalent designs, including mosaic or nanoparticle-based platforms, simultaneously present multiple antigenic variants, promoting the expansion of cross-reactive B-cell clones through affinity maturation. While chimeric approaches offer greater control over immune focusing, multivalent strategies typically provide broader coverage, albeit with increased manufacturing and formulation complexity.

The development of a universal SARS-CoV-2 vaccine should also leverage the extensive immunological data generated from vaccinated but infection-naïve individuals. Analyses of their humoral and cellular immune responses reveal durable and partially cross-reactive protection, offering valuable insights into correlates of breadth and longevity. Integrating these data into antigen design pipelines may accelerate the identification of conserved epitopes and facilitate the development of vaccines capable of protecting against a wider spectrum of coronaviruses.

The relatively weak immunogenicity of recombinant protein antigens further necessitates the use of advanced delivery systems and adjuvants. Nanoparticle carriers enhance antigen density and multivalent presentation, thereby amplifying B-cell activation and promoting coordinated humoral and cellular responses. In parallel, next-generation adjuvants that activate dendritic cells, promote cytokine production, and facilitate efficient cross-presentation to CD8^+^ T cells are essential for strengthening cellular immunity and establishing durable immune memory. These components are therefore integral, not ancillary, to achieving broad and long-lasting protection.

Finally, the route of immunization adds an additional layer of complexity. Mucosal vaccination has the potential to induce local immune responses at viral entry sites, complementing systemic immunity elicited by parenteral vaccines. However, mucosal immunity alone may be insufficiently durable, emphasizing the need for strategic combinations of systemic and mucosal vaccination approaches.

Taken together, the pursuit of a truly universal SARS-CoV-2 vaccine should be viewed not as a single antigenic solution but as a multidimensional design challenge that integrates conserved antigen targeting, balanced B- and T-cell immunity, with particular emphasis on CD8^+^ T-cell responses, advanced vaccine platforms, and optimized delivery routes. Although complete sterilizing immunity against all variants may remain unrealistic, an achievable and impactful goal is the development of vaccines that confer durable, cross-variant protection against severe disease and transmission. Ultimately, the realization of a truly universal SARS-CoV-2 vaccine will depend not on incremental optimization of individual platforms, but on the precise identification, rational integration, and effective presentation of conserved, functionally constrained epitopes capable of engaging complementary arms of the immune system.

## Figures and Tables

**Table 1:** Comparison of antigen design strategies and vaccine platforms for broad-spectrum and universal SARS-CoV-2 vaccination.

Antigen Strategy	Platform	Model System(s)	Breadth Endpoint	Development Stage	Key Limitation(s)	Ref.
Mosaic RBD (multiple variant RBDs)	Protein nanoparticle; ferritin nanoparticle	Mouse, NHP	Broad neutralization of SARS-CoV-2 VOCs (variants of concern); partial cross-neutralization of SARS-CoV-1	Preclinical/Phase 1	Spike-focused; potential sensitivity to future RBD divergence	[59]
Multivalent RBD assemblies (heterologous dimers/trimers/dodecamers)	mRNA-LNP; protein nanoparticle	Mouse	Neutralization of Alpha, Beta, Gamma, Delta, Omicron	Preclinical	Antigen complexity; immune imprinting risk	[57,68,69,70]
Conserved S2 epitopes (FP, HR1–HR2, stem-helix)	Protein subunit; nanoparticle display	Mouse, NHP	Cross-variant binding; partial cross-sarbecovirus reactivity	Preclinical	Low intrinsic immunogenicity; subdominant epitopes	[56,61]
Hybrid antigens (variable RBD + conserved regions)	Recombinant protein; mRNA-LNP; nanoparticle	Mouse, hamster	Broad neutralization across SARS-CoV-2 variants	Preclinical	Immunodominance of variable regions; design complexity	[58,66,67,68,69,70]
T-cell epitope cassette (CD4^+^/CD8^+^ conserved epitopes)	mRNA-LNP; DNA; viral vector	Mouse, hamster	Cross-variant cellular immunity; protection from severe disease	Preclinical	Limited sterilizing immunity; HLA restriction	[58]
Combined B- and T-cell multi-epitope vaccines	Protein subunit; nanoparticle	Mouse, hamster	Protection against severe disease across VOCs	Preclinical	Optimization of epitope composition required	[48,58]
Ferritin-based multivalent nanoparticles (SpFN, RFN, DCFHP)	Self-assembling ferritin nanoparticle + adjuvant	Mouse, NHP, human	Broad neutralization of VOCs; partial pan-sarbecovirus activity	Phase 1	Manufacturing scale-up; durability under evaluation	[60,61,62,63,64]
Authorized mucosal vaccines (adenoviral or protein-based)	Intranasal viral vector or protein	Human	Mucosal IgA induction; limited variant coverage	Emergency/ conditional authorization	Limited real-world effectiveness data	[81,82]
Live attenuated mucosal vaccines (e.g., IBIS)	Intranasal attenuated virus	Mouse, hamster	Protection vs. SARS-CoV-2 variants and SARS-CoV-1; transmission blocking	Preclinical	Regulatory complexity; safety monitoring	[91]

The table synthesizes representative vaccine candidates and platform technologies discussed in Section 3.3 and Section 3.4 (nanoparticle-based and hybrid/multivalent antigen strategies) as well as Section 4 and Section 5 (adjuvant systems and mucosal vaccine approaches). Strategies are compared with respect to antigen composition, delivery platform, experimental model, breadth of immune protection (including cross-variant, cross-sarbecovirus, and transmission-blocking endpoints), developmental stage, and key limitations relevant to the development of universal coronavirus vaccines.

## Data Availability

No new data were created or analyzed in this study. Data sharing is not applicable to this article.
